# Evidence-based practice profiles among bachelor students in four health disciplines: a cross-sectional study

**DOI:** 10.1186/s12909-018-1319-7

**Published:** 2018-09-14

**Authors:** Anne Kristin Snibsøer, Birgitte Graverholt, Monica Wammen Nortvedt, Trond Riise, Birgitte Espehaug

**Affiliations:** 1 Centre for Evidence-Based Practice, Faculty of Health and Social Sciences, Western Norway University of Applied Sciences, Postbox 7030, 5020 Bergen, Norway; 2 Accident and Emergency Department, Bergen Municipality, Postbox 7700, 5020 Bergen, Norway; 3 Department of Global Public Health and Primary Care, University of Bergen, Postbox 7804, 5020 Bergen, Norway

**Keywords:** Evidence-based practice, Students, Nursing, Occupational therapy, Physiotherapy, Radiography, Attitude, Knowledge, Behaviour

## Abstract

**Background:**

Despite the recognition of integrating evidence-based practice (EBP) in educational programs, there is limited research about bachelor students’ EBP profiles (EBP knowledge, attitudes and behaviour) in the health disciplines nursing, occupational therapy, physiotherapy and radiography. The aim of this study was to assess EBP profiles among bachelor students in health disciplines, and explore differences between health disciplines, educational institutions, students’ assessment of EBP teaching and expectations of EBP performance.

**Methods:**

A survey using the ‘Evidence-Based Practice Profile - Norwegian version’ (EBP^2^-N) was conducted among final year bachelor students in health disciplines from four educational institutions. The questionnaire consisted of five domains (Relevance, Terminology, Confidence, Practice and Sympathy) and assessed the five steps of EBP. We performed regression analyses to analyse mean differences in domain scores between health disciplines, Cohen’s *d* to illustrate the magnitude of the largest difference in each domain, Omega squared to describe portion of variance in domain scores, and Spearman’s rho (r_s_) to assess the monotonic relationship between EBP^2^-N domains and assessment of EBP teaching and expectations of EBP performance, respectively.

**Results:**

Students reported highest overall mean score for Relevance, with an estimated standardized mean of 81.2 (CI 95% = 80.4–82.0). The other EBP^2^–N domains had estimated standardized means of 54 and less. Statistically significant differences (*p* < 0.03) between health disciplines were observed for all domains. The largest mean difference was found for Relevance with highest score for occupational therapy and lowest for radiography, with an estimated Cohen’s *d* of 1.11. Moderate positive associations were observed between Relevance scores and students’ assessment of EBP teaching (r_s_ = 0.31), and expectations of EBP performance from teachers (r_s_ = 0.36). We also observed a moderate positive correlation between Confidence and students’ assessment of EBP teaching (r_s_ = 0.46).

**Conclusion:**

Bachelor students in health disciplines found EBP relevant, but revealed low understanding of EBP terminology, low confidence with EBP skills, and low use of EBP in clinical situations. We observed differences in EBP profiles between health disciplines and between educational institutions. The differences in scores raise questions about the understanding of EBP within disciplines, and the complexity of EBP in educational settings.

**Electronic supplementary material:**

The online version of this article (10.1186/s12909-018-1319-7) contains supplementary material, which is available to authorized users.

## Background

Evidence-based practice (EBP) is a systematic approach to clinical decision-making which incorporates the current best available evidence from research and clinical expertise with the values and preferences of health service users, within a context of available resources [1]. There is an expectation that health professionals apply knowledge and skills in their professional work that is based on the best available evidence, use evidence to inform practice, and constantly strive to use evidence-based approaches to improve health system performance [2–4]. However, studies have demonstrated that even though health professionals hold positive attitudes towards EBP there is a lack of EBP utilization in practice [5, 6]. A number of barriers towards EBP have been described, including lack of time, lack of availability and accessibility of research, lack of cultures that recognize EBP performance, and lack of EBP knowledge and skills among health professionals [6–9].

New demands in healthcare impose changes in healthcare education and training. International federations of healthcare acknowledge that the teaching of EBP skills and research methodology should be integrated in healthcare educations [10–12]. Moreover, the Lancet commission report *Education of health professionals for the twenty-first century* propose a shift in healthcare training towards producing enlightened change agents [13]. By emphasizing the importance of lifelong learning, and recommending transformative learning that embraces the transfer from facts memorization to searching, analysis and synthesis of information for decision-making, the report supports the need for EBP knowledge and skills. The progressive focus on EBP is also included in Norwegian healthcare educational policies, which recommend mandatory teaching in EBP for all bachelor students in health disciplines [14].

An international curriculum framework for EBP, with recommendations for EBP teaching and education, was first described in the Sicily statement on EBP [1]. According to this consensus statement, EBP should be a basic and essential component of curricula. The EBP teaching should be based on the five step model of EBP, which typically involves the ability to formulate a research question, conduct a systematic search for literature, critically appraise the evidence, apply the evidence into clinical practice and evaluate performance [1]. To promote lifelong learning, the basic skills of EBP should be taught early and integrated in curricula through all study years [15]. In addition, it is recommended that the teaching and learning strategies are multifaceted, clinically integrated and include knowledge and skills assessment [16].

Despite the recognition of integrating EBP in healthcare educational programs, there is diversity in how bachelor students in health disciplines perceive EBP. Results of previous surveys assessing EBP knowledge, attitude and behaviour among healthcare students have been inconsistent [17–24]. The studies have applied different instruments, mainly to students within a single profession. Only one instrument has been explicitly developed to cover the range of EBP domains likely to change as a result of education and training across health professions [25]. Moreover, one study has used this instrument to compared EBP profiles (EBP knowledge, attitude and behaviour) between students in the allied health professions [22]. No previous studies have compared EBP profiles between bachelor students in nursing and the allied health professions. The aim of this study was to assess EBP profiles among bachelor students in health disciplines, and explore differences between health disciplines, educational institutions, students’ assessment of EBP teaching and expectations of EBP performance.

## Methods

We conducted an analytic cross-sectional study among Norwegian final year bachelor students in health disciplines during spring 2015.

### Setting

In Norway, a bachelor’s degree is the entry-level to practice as a nurse, occupational therapist, physiotherapist or radiographer. The 3 year bachelor programs constitute 180 European Credit Transfer and accumulation System (ECTS), distributed on theoretical and clinical studies [26] (Table 1). At the time of data collection, full-time education in healthcare were offered at 21 University Colleges and four Universities distributed across the country (Table 1).

**Table 1:** Distribution of ECTS in National Curricula and educational institutions for Norwegian bachelor programs in healthcare

	Distribution of 180 ECTS			Bachelor programs at
Health discipline	Theoretical studies	Skills training at school	Placement in clinical practice	University (U) University College (UC)
Occupational therapy	105	15	60	1 U + 5 UC
Physiotherapy	105	30	45	1 U + 3 UC
Radiography	111	9	60	1 U + 5 UC
Nursing	90	15	75	4 U + 21 UC

Norwegian National Curricula set standards for healthcare bachelor programs by describing overall aims, content and required competences upon completion of programs [26]. The National Curricula for all healthcare programs consists of a profession-specific content (150 ECTS) and a common content (30 ECTS). The common content includes core competences that are shared by all bachelor programs in healthcare [26], and are often taught interdisciplinary. Based on the National Curricula, higher educational institutions develop their own curricula, which includes information about their programs’ aims, core competences, learning outcomes and contents, as well as the organization, progression and facilitation of programs [27]. A National Agency for Higher Education (NOKUT) accredits and monitors the quality of these programs [28].

All bachelor programs represented in this study addressed EBP in their curricula. A document review was performed to indicate when EBP was stated in the curricula learning outcomes and content (Table 2). Two people (first author and a research assistant) carried out the document review. They searched all curricula for the word *evidence-based practice* and terms related to the five-step model of EBP (ask, acquire, appraise, apply and assess). To determine if the terms were related to EBP training, we judged whether the use of each term within the sentence was specific to EBP and not to research in general.

**Table 2:** EBP explicit (E), implicit (I) or not mentioned (−) in curricula (2012–2015)

		EBP in programs’ curricula						
			Semester					
School	Health discipline	Overall aim	1	2	3	4	5	6
A	Occupational	E	E	E	E	E	E	E^a^
	Physiotherapy	E	E	–	E	E	–	I^b^
	Radiography	E	E	E	I	E	E	I
	Nursing	E	–	–	–	E	–	I
B	Occupational	E	–	–	E	I	E	E
	Radiography	E	E	–	I	–	I	E
	Nursing	E	E	E	E	E	E	E
C	Occupational	E	–	–	–	I	E	E
	Physiotherapy	E	–	–	–	I	I	E
	Radiography	E	–	I	E	–	E	I
	Nursing	E	I	I	E	–	E	E
D	Occupational	E	I	–	–	E	–	E
	Physiotherapy	E	I	–	–	–	I	–
	Radiography	I	I	–	–	–	–	I
	Nursing	E	I	–	I	E	I	I

^a^ E = EBP explicitly mentioned by word

^b^ I = EBP implicitly mentioned by elements of the EBP steps (ask, acquire, appraise, apply or assess), but indefinite if curricula reflected EBP or research in general

### Participants

The participants in our study consisted of final year bachelor students in nursing, occupational therapy, physiotherapy and radiography. The convenience sample was recruited from the four Norwegian educational institutions that, at the time of data collection, offered all of these bachelor programs fulltime.

**Fig. 1:**
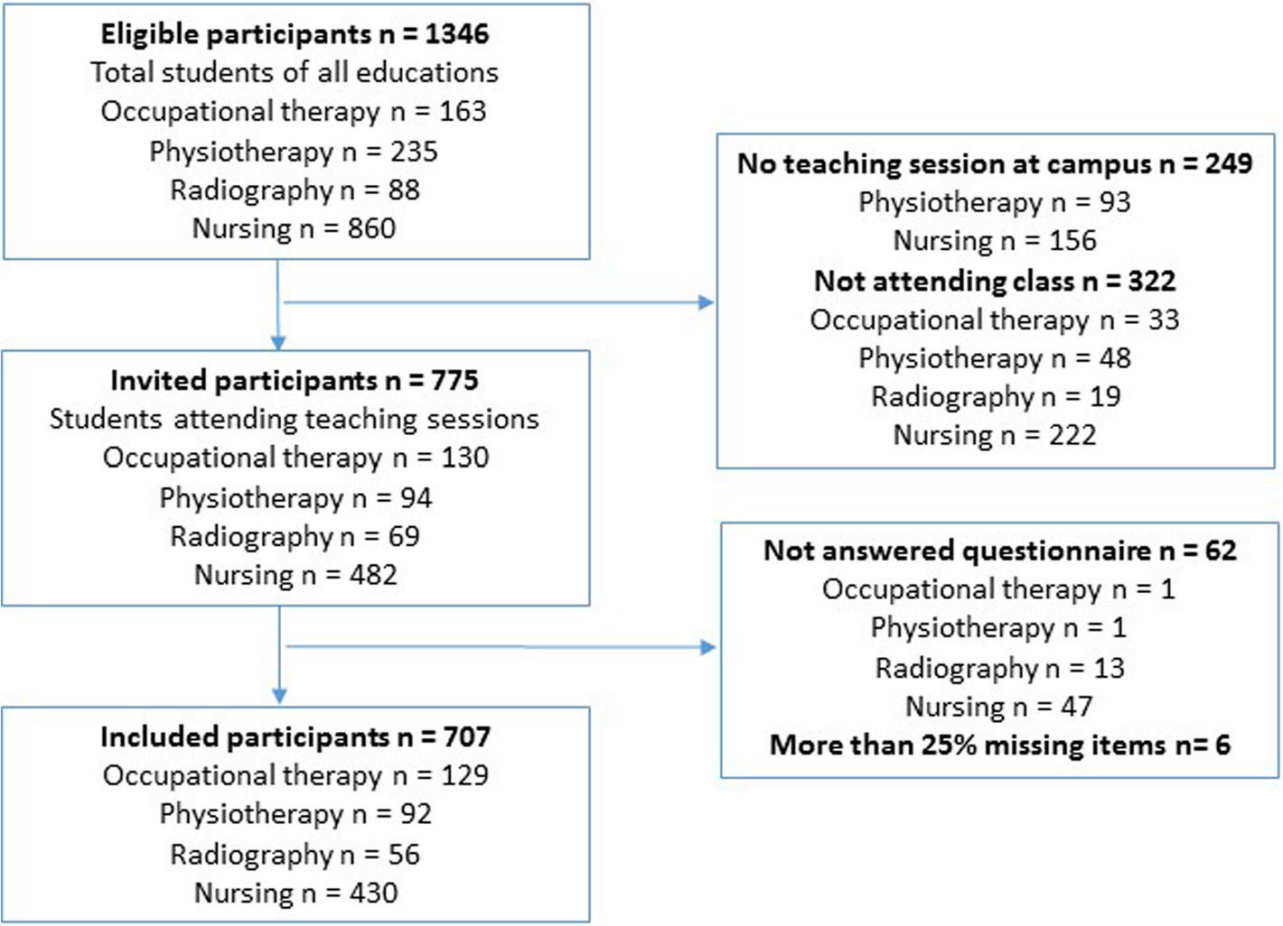
Flow diagram of included participants

Students enrolled in 16 programs at four educational institutions were eligible to participate (*n* = 1346). We collected data at teaching sessions with expected high student attendance during spring term 2015. One program in physiotherapy and two classes of nursing were excluded as they had no teaching sessions at campus during the data collection period (*n* = 249). Students absent during the sessions (*n* = 322) were not included. Thus, 775 students were invited to participate (Fig. 1).

Data were collected from March – June 2015. The students received information about the study on the students’ online learning platform 2 days before data collection. During the teaching session, a researcher handed out and collected the paper-based questionnaire. The teaching sessions varied in content and included a range of topics.

### Measurement

We used the Norwegian version of the Evidence-Based Practice Profile (EBP^2^) questionnaire [25, 29], a self-reported questionnaire that examines self-perceived EBP knowledge, attitude and behaviour. The questionnaire is trans-professional, assesses the five steps of EBP and incorporates elements of EBP that are likely to change as a result of education, training and exposure over time. It consists of 73 items, whereof 58 items relate to the five domains of Relevance, Terminology, Confidence, Practice and Sympathy. The domains explore the value, emphasis and importance participants place on EBP (Relevance, 14 items), the understanding of common research terms (Terminology, 17 items), the perception of ability with EBP skills (Confidence, 11 items), the use of EBP in clinical situations (Practice, 9 items), and the perception of compatibility of EBP with professional work (Sympathy, 7 items) [25]. The domain of Sympathy has negatively phrased items, which need to be reversed before analysis. Each item was weighted equally within the domain.

The non-domain items included 15 educational items and six demographic characteristics, including gender, age, health discipline, educational institution, previous bachelor education and percentage of paid work besides studies. A subset of the educational items assessed the participants’ assessment of EBP teaching (4 items) and assumed expectations of EBP performance from teachers and clinical instructors (2 items).

All items were scored on a 5-point Likert scale, with a minimum score of 1 and a maximum score of 5 per item. A summary score for each domain was calculated by summing the scores of the items within the domain. In addition, we calculated standardized summary scores on a scale from 0 to 100 and standard summary scores (Z-scores). Respondents with more than 25% missing items on non-demographic items were excluded from further analysis. Respondents with more than 20% missing values on one domain were excluded from analysis of that specific domain. For respondents with missing items of 20% or less within a domain we substituted the missing scores with the mean of the other items in the domain.

The Evidence-Based Practice Profile – Norwegian version (EBP^2^-N) has been translated, culturally adapted and psychometric tested [29]. EBP^2^-N was found valid and reliable for the domains Relevance, Terminology and Confidence, and responsive to change for all domains, except Sympathy. The authors recommended further linguistic improvement. Thus, before the questionnaire was used in our survey, the EBP^2^-N was reviewed and imprecise items (*n* = 12) were revised by an expert panel. The confirmatory factor analysis (CFA) showed the same results as in the validation study [29]. Cronbach’s alpha for the five domains ranged from 0.69 (Sympathy) to 0.90 (Confidence) (Additional file 1).

### Statistical analysis

A power analysis informed that at least 64 students should be included from each health discipline to detect a standardized mean difference (effect size) of 0.5 as statistically significant (two-sided hypothesis test; α = 0.05) with a power of 80%. We chose to include all available students to ensure adequate numbers for multiple regression analyses and for subgroup analyses.

Descriptive analyses were applied for demographic characteristics. The Chi-square test and one-way analysis of variance (ANOVA) were performed to test for distributional differences among the four health disciplines (nursing, occupational therapy, physiotherapy and radiography), gender, educational institutions (school A, B, C and D), previous bachelor education (yes, no), paid work in addition to studies (0%, 1–20%, 21–50% and > 50% of a full time employment of 37.5 h per week) and age (in years).

Differences in mean domain scores between health disciplines and between educational institutions were analysed by ANOVA. Linear regression analyses were performed to examine the extent health discipline predicted domain scores. In the model, we controlled for possible confounding by the variables educational institution, gender, age, previous bachelor education and paid work. We used the original scores as outcome variables for these analyses. Nursing was defined as the reference category, and the estimated regression coefficients (beta) represent mean differences in scores for the allied health professions compared to nursing. Goodness-of-fit was assessed by the adjusted coefficient of determination (R^2^).

To illustrate the magnitude of the difference we calculated Cohen’s *d* (standard deviation units) as the difference between the highest and lowest mean within each domain divided by the pooled standard deviation. In addition, we calculated Omega squared (ω^2^) to describe the proportion of variance in domain scores that could be explained by health discipline. Spearman’s rho (r_s_) was used to assess the monotonic relationship between the EBP^2^-N domains, and students’ assessment of EBP teaching and students’ assumed expectations from teachers of EBP performance, respectively.

Cohen’s *d* (standard deviation units) was considered small if 0.2, medium if 0.50 and large if 0.80 [30]. Spearman’s rho was interpreted as small if < 0.30, medium if 0.31–0.49 and large if ≥0.50 [30].

*p*-values less than 0.05 indicated statistical significance. The statistical software IBM SPSS Statistics version 22 [31] and *R* [32] were used for the statistical analyses.

## Results

Of the 775 students who attended the teaching sessions, 713 (92%) answered the questionnaire. Six respondents had more than 25% missing items, allowing 707 responses to be included in the analysis (Fig. 1).

The respondents were students in occupational therapy (18%), physiotherapy (13%), radiography (8%) and nursing (61%) (Table 3). The mean age was 25.1 (SD ± 4.8) years (range 20–56 years). The proportion of men was highest among radiography students (32%) and lowest among nursing students (9%). Most participants (91%) had no previous bachelor education and the majority (84%) had paid work besides their studies.

**Table 3:** Characteristics of participants for the total sample and for each health discipline

	Total (*n* = 707)	Occupat therapy (*n* = 129)	Physio-therapy (*n* = 92)	Radio-graphy (*n* = 56)	Nurse (*n* = 430)	*p*-value
	n (%)	n (%)	n (%)	n (%)	n (%)	
Gender^a^						*p* < 0.001^b^
Female	599 (85)	101 (78)	71 (77)	38 (68)	389 (91)	
Male	106 (15)	28 (22)	21 (23)	18 (32)	40 (9)	
Educational institution						*p* < 0.001^b^
School A	162 (23)	29 (23)	37 (40)	13 (23)	83 (19)	
School B	197 (28)	38 (30)	0	11 (20)	148 (34)	
School C	244 (35)	52 (40)	40 (44)	22 (39)	130 (30)	
School D	104 (15)	10 (8)	15 (16)	10 (18)	69 (16)	
Previous bachelor education^a^						*p* = 0.2^b^
Yes	56 (8)	5 (4)	10 (11)	5 (9)	36 (9)	
No	643 (91)	124 (96)	82 (89)	50 (91)	387 (91)	
Work in addition to studies^a^						*p* < 0.001^b^
0%	103 (15)	28 (22)	20 (22)	10 (18)	45 (11)	
1–20%	399 (56)	62 (48)	55 (60)	23 (41)	259 (61)	
21–50%	179 (25)	38 (30)	15 (17)	18 (32)	108 (25)	
> 50%	23 (3)	1 (1)	1 (1)	5 (9)	16 (4)	
Age						*p* = 0.06^c^
N	701	129	92	55	425	
Mean (SD)	25.1 (4.8)	25.7 (5.2)	24.0 (2.6)	25.2 (4.5)	25.2 (5.0)	
Min - Max	20–56	21–50	21–38	20–43	21–56	

^a^Number of missing values was 5 for gender, 10 for previous bachelor education and 5 for work in addition to studies

^b^analyzed by Chi-square

^c^analyzed by one-way ANOVA

The highest overall mean score was observed for Relevance, with an estimated standardized mean of 81.2 (CI 95% = 80.4–82.0). The other EBP^2^-N domains had estimated standardized means of 54 and less (Table 4).

**Table 4:** Mean level of bachelor students’ (*n* = 707) EBP^2^-N scores and test of mean differences across four health disciplines

EBP^2^-N domains (max value)	Total sample		Health disciplines			
	Original scale	Standardized^a^	*F*	P	Cohen’s *d*	ω^2^
	Mean (95% CI)	Mean (95% CI)				
Relevance (70)	59.5 (59.0–59.9)	81.2 (80.4–82.0)	15.14	< 0.001	1.11	0.06
Terminology (85)	47.0 (46.2–47.9)	44.1 (42.9–45.4)	8.60	< 0.001	0.69	0.03
Confidence (55)	34.8 (34.3–35.3)	54.1 (52.9–55.3)	8.95	< 0.001	0.44	0.03
Practice (45)	23.8 (23.4–24.2)	41.0 (39.9–42.1)	5.08	0.002	0.60	0.02
Sympathy (35)	21.8 (21.5–22.0)	52.7 (51.8–53.7)	3.03	0.03	0.43	0.01

^a^0–100 scale, calculated as (observed score – min domain score)*100 / (max domain score – min domain score)

**Fig. 2:**
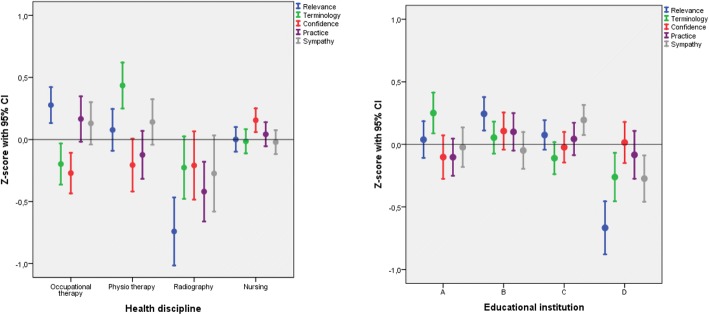
Z-score values for the EBP^2^-N domains by health discipline and educational institution

Differences in mean scores among health disciplines were small, but statistically significant for all domains (*p* ≤ 0.03) (Table 4 and Additional file 2). The largest difference was found for Relevance, with highest score for occupational therapy and lowest for radiography, with an estimated Cohen’s d of 1.11 (Table 4). Students in radiography consistently reported low mean domain scores, with mean Z-scores below the average for all EBP^2^-N domains (Fig. 2). Domain Z-scores among health disciplines varied from − 0.77 (95% CI -1.04 – -0.5) (Relevance) for radiograph students to 0.43 (95% CI 0.25–0.62) (Terminology) for physiotherapy students (Fig. 2). The difference between health disciplines persisted after adjustment for educational institution, gender, age, previous bachelor education and work in addition to studies (Additional file 3).

The mean EBP^2^-N scores differed significantly between educational institutions for three domains (Relevance *p* < 0.001, Terminology *p* < 0.001 and Sympathy *p* = 0.001). The largest difference was found for Relevance, with an estimated Cohen’s d of 0.86. Among educational institutions, school D reported mean Z-scores below the average for all domains, except Confidence. Domain Z-scores among educational institutions varied from − 0.66 (95% CI -0.87 – -0.45) (Relevance) for school D to 0.27 (95% CI 0.10–0.43) (Terminology) for school A (Fig. 2).

A medium positive correlation was observed between Relevance and students’ assessment of EBP teaching (r_s_ = 0.31, *n* = 693, *p* < 0.001), between Relevance and students’ assumed expectations from teachers of EBP performance (r_s_ = 0.36, *n* = 696, *p* < 0.001), and between Confidence and assessment of EBP teaching (r_s_ = 0.46, *n* = 691, *p* < 0.001) (Additional file 4).

## Discussion

This is the first study to assess EBP profiles among bachelor students in nursing and allied health professions. We found that bachelor students across health disciplines found EBP relevant, but revealed low understanding of EBP terminology, low confidence with EBP skills, and low use of EBP in clinical situations. Differences in domain specific results were observed between health disciplines and between educational institutions. In addition, we found that students with positive assessments of EBP teaching also perceived EBP as more relevant, and they were more confident with their EBP skills. Moreover, students who perceived high EBP expectations from teachers found EBP more relevant.

The high overall mean score for Relevance indicates that the respondents placed high values, emphasis and importance on EBP. Our findings for this domain was equivalent to and slightly higher than findings from previous studies using the same instrument [20, 22, 33, 34]. Although we did not examine EBP exposure specifically, our results for Relevance corresponded to findings among Australian students with more than 20 hours of EBP exposure and to Australian students who had undertaken formal EBP training, including stand-alone courses and integration of EBP in professional theoretical courses and supervised clinical practice [20, 22, 33, 34].

Our students’ positive perception of Relevance did not translate into the other EBP^2^-N domains. Our findings for Terminology and Confidence were lower than previously reported for students undertaking EBP training [20, 33, 34]. For these domains, our results were in line with findings from students with less than 20 hours of EBP exposure [22]. For the domains of Practice and Sympathy, our findings were more consistent with previous studies [20, 22, 33, 34], although we for Practice also observed lower mean values as compared to Australian students with formal EBP training [20, 34]. However, taken into account the differences in samples, educational systems, EBP exposure and training, our comparisons of findings should be interpreted with caution. Nevertheless, it is of interest to observe the EBP profiles in our sample in light of the findings from students in different contexts.

One explanation for our positive result of Relevance may be the progressive focus on EBP in Norwegian higher education, healthcare policies and media. We observed that students who perceived higher expectations of EBP from teachers also placed higher value, emphasis and importance on EBP. Media, referring governmental policies and national discussions focusing on an evidence-based society, may have functioned as reinforcement of the students’ academic exposure and added value to how relevant they viewed EBP. With external expectations and self-reported measurements there is always a risk of providing socially desirable responses rather than actual attitudes. However, we found a moderate association between perceived EBP expectations and Relevance, and this was not evident for the other EBP^2^-N domains. It is therefore likely that multiple exposures to EBP may have influenced our participants’ positive attitudes towards EBP.

The students in our study reported low scores for Terminology and Confidence. These findings might indicate that a three-year bachelor program is too short to incorporate EBP knowledge and skills in healthcare educational programs. Another plausible explanation could be lack of competence in teaching EBP among faculty at the bachelor’s level. Although the teaching and learning of EBP in Norwegian higher education is upcoming, the tradition of teaching has centred on how to conduct research rather than how to use the best evidence to inform practice. Faculty may hold positive attitudes towards EBP and be knowledgeable in basic methodology, but lack knowledge and skills in the EBP process of appraising and applying evidence in practice [35–37]. However, knowledge and skills in research methodology does not necessarily translate into supportive attitudes towards EBP, knowledge of the EBP process, or skills in acquiring and appraising evidence [36, 38]. We found that students with positive assessments of EBP teaching found EBP more relevant and were more confident with EBP skills. Hence, to enhance educational cultures that ensure students’ competences and appreciation of EBP it is essential to understand faculty’s knowledge, attitudes, and practice of teaching EBP, and to upskill faculty in the EBP process [36–38].

Our results revealed small, but statistically significant differences in domain scores between disciplines. We did not identify any systematic patterns in the EBP profiles, and it is challenging to find plausible explanations for the physiotherapy students’ higher Terminology scores, the nursing students’ higher Confidence scores and the radiography students’ lower Relevance scores. In line with our results, McEvoy et al. [22] also observed higher scores for Terminology among physiotherapy students and lower EBP^2^ domain scores for students in medical radiation.

A possible explanation for the physiotherapy students’ higher Terminology scores might be that the physiotherapy program has a stronger focus on research and methodology, as physiotherapists frequently use tests based on quantitative studies for diagnosis and treatment. It is also plausible that our nursing programs’ extensive clinical placement periods may explain the nursing students’ higher Confidence scores. The nursing profession in Norway has been highly representative in postgraduate EBP programs [39], and there is a possibility that clinical nurses engaged in EBP may have encouraged their students’ confidence in EBP.

One can only speculate why the radiography students reported lower scores for Relevance, and mean scores below the average for all other EBP^2^-N domains. It may be argued that, compared to the other included health disciplines, radiographers are more involved in diagnosis than treatment, they participate less in the daily care of patients and their job entails technical tests ordered by other health professionals. Upton et al. [40] have previously illuminated that EBP might be perceived as less relevant for professionals engaged in diagnosis rather than treatment, and in workplace cultures that expect adherence to sets of rules rather than questioning of practice. Thus, one might question if less independency in clinical decisions might be a reason for lower EBP perceptions.

During our curriculum review, we found fragmented, imprecisely and implicitly formulated learning outcomes related to EBP across all programs. Interestingly, students attending the educational institution (school D) with less integration of EBP in curricula reported lower scores for Relevance, and mean standard scores below the average for most of the other domains. We are aware that our document review does not truly reflect our students EBP exposure and experience, and our study was not designed to capture EBP teaching and learning approaches. Still, inadequate descriptions of EBP learning outcomes in curricula have been observed in other studies [41], and McEvoy et al. [42] have argued that accreditation bodies should recognize EBP in accreditation documents to prioritize the integration of EBP into entry-level programs. Thus, to ensure explicit EBP competences upon completion of bachelor programs, regular reviews of EBP learning outcomes in programs’ curricula is needed. In line with findings from systematic reviews [16] and recommendations from experienced educators in EBP [1], educational institutions should develop comprehensive curricula where EBP teaching and learning is integrated throughout entire study programs, allowing repetition, consolidation and application of EBP knowledge and skills.

### Strengths and limitations

Some limitations to this study have already been presented throughout the discussion, including lack of information about the students’ actual EBP teaching and learning. In addition, analysis of structural validity of the EBP^2^-N did not confirm the original five-factor model. Subsequently, the results of Sympathy and Practice should be interpreted with caution, as structural validity was not confirmed for these domains.

The response rate was high among students attending teaching sessions. Still, the smaller sample size of radiography students is a limitation to this study. Additionally, a large proportion of eligible students was not included in the study, and we lacked information to analyse non-responders. The allied health programs were well represented in the study. This was not the case for nursing students, where a limited proportion of educational institutions were included. Still, we considered diversity by including educational institutions located throughout the country, and by collecting data during teaching sessions with various topics. By including students from four health disciplines, attending various teaching sessions at four different educational institutions, we have provided insight into differences in EBP profiles at one point of time across a variety of Norwegian bachelor students in health disciplines.

In the analyses we adjusted for a range of possible confounders. A substantial amount of variability in the outcome measures was unaccounted for indicating that other, possible context specific, factors could have resulted in a better model fit.

## Conclusion

Bachelor students in health disciplines found EBP relevant, but revealed low understanding of EBP terminology, low confidence with EBP skills, and low use of EBP in clinical situations. We observed differences in EBP profiles between health disciplines and between educational institutions. The differences in scores raise questions about the understanding of EBP within disciplines, and the complexity of EBP in educational settings. Our findings underline that bachelor students in health disciplines are not equally prepared for EBP.

## Additional files

Additional file 1: Cronbach’s Alpha for the EBP^2^-N domains. The table provides Cronbach’s Alpha results as a measure of the reliability for the five EBP^2^-N domains. (PDF 398 kb)

Additional file 2: Descriptive statistics for EBP^2^-N domains by participants’ characteristics (*n* = 707). The table provides the mean score values for the five EBP^2^-N domains by health disciplines, educational institutions, gender, previous bachelor education, work in addition to studies and age. Group differences were analysed by ANOVA and t-tests. (PDF 575 kb)

Additional file 3: Estimated differences in mean EBP^2^-N domain scores between health disciplines (n = 707). The table provides differences in mean EBP^2^-N domain scores estimated by simple and multiple linear regression for health disciplines with nurses as the reference group. Goodness-of-fit was assessed by the adjusted coefficient of determination (R^2^). (PDF 74 kb)

Additional file 4: The relationship between EBP^2^-N domains, assessment of EBP teaching and expectation of EBP performance. The table provides the results of the relationship of the five EBP^2^-N domains, and students’ assessment of EBP teaching and students’ assumed expectations from teachers of EBP performance, respectively, estimated by Spearman’s rho (r_S_). (PDF 314 kb)

Additional file 5: Dataset. The file provides the non-identifying data used in the current study (all data except gender and age). (XLSX 196 kb)

## Abbreviations

ANOVA: Analysis of variance
CFA: Confirmatory factor analysis
CI: Confidence intervals
EBP: Evidence based practice
EBP^2^: Evidence-based practice profile
EBP^2^-N: Evidence-based practice profile – Norwegian version
ECTS: European credit transfer and accumulation system
r_s_: Spearman’s rho
Z-scores: Mean standard score
