# Supplementary material for: Evidence-based practice profiles among bachelor students in four health disciplines: a cross-sectional study
Source: BMC Med Educ. 2018 Sep 14;18:210. doi: 10.1186/s12909-018-1319-7 (PMC6137748; doi:10.1186/s12909-018-1319-7)
Supplement: Supplementary file 1 — Cronbach’s Alpha for the EBP2-N domains. The table provides Cronbach’s Alpha results as a measure of the reliability for the five EBP2-N domains. (PDF 398 kb) [file 12909_2018_1319_MOESM1_ESM.pdf]

Additional file 1. Cronbach's Alpha for the EBP^2-N domains.

Domain	N of items	N	Cronbach's Alpha
**Relevance**	14	677	0.86
**Terminology**	17	657	0.89
**Confidence**	11	682	0.90
**Practice**	9	659	0.83
**Sympathy**	7	675	0.69
