# Supplementary material for: Evidence-based practice profiles among bachelor students in four health disciplines: a cross-sectional study
Source: BMC Med Educ. 2018 Sep 14;18:210. doi: 10.1186/s12909-018-1319-7 (PMC6137748; doi:10.1186/s12909-018-1319-7)
Supplement: Supplementary file 2 — Descriptive statistics for EBP2-N domains by participants’ characteristics (n = 707). The table provides the mean score values for the five EBP2-N domains by health disciplines, educational institutions, gender, previous bachelor education, work in addition to studies and age. Group differences were analysed by ANOVA and t-tests. (PDF 575 kb) [file 12909_2018_1319_MOESM2_ESM.pdf]

Additional file 2. Descriptive statistics for EBP[2]-N domains by participants' characteristics (n=707).

	EBP[2]-N domains				
	Relevance (70)	Terminology (85)	Confidence (55)	Practice (45)	Sympathy (35)
	mean (SD)	mean (SD)	mean (SD)	mean (SD)	mean (SD)
**Health discipline (n)**					
Occupational (129)	61 (5)[a]	45 (11)[a]	33 (7)[a]	25 (6)[a]	22 (3)[a]
Physiotherapy (92)	60 (5)[b]	52 (10)[abc]	33 (7)[b]	23 (5)	22 (3)
Radiography (56)	55 (6)[abc]	45 (10)[b]	33 (7)[c]	22 (5)[ab]	21 (4)[a]
Nursing (430)	59 (6)[ac]	47 (11)[c]	36 (7)[abc]	24 (5)[b]	22 (3)
p value*	<0.001	<0.001	<0.001	0.002	0.03
**Educational institution (n)**					
School A (162)	60 (6)[a]	50 (12)[a]	34 (8)	23 (5)	22 (3)
School B (197)	61 (6)[b]	48 (10)[b]	35 (7)	24 (6)	22 (4)
School C (244)	60 (6)[c]	46 (11)[a]	35 (7)	24 (5)	22 (3)[a]
School D (104)	55 (7)[abc]	44 (11)[ab]	35 (6)	23 (5)	21 (3)[a]
p value*	<0.001	<0.001	0.4	0.3	0.001
**Gender (n)**					
Female (599)	59 (6)	46 (11)	35 (7)	24 (5)	22 (3)
Male (106)	60 (6)	52 (10)	36 (7)	24 (5)	23 (4)
p value**	0.8	<0.001	0.06	0.8	0.01
**Previous bachelor education (n)**					
Yes (56)	60 (6)	52 (10)	37 (6)	25 (5)	22 (4)
No (643)	59 (6)	47 (11)	35 (7)	24 (5)	22 (3)
p value**	0.4	<0.001	0.06	0.1	0.7
**Work in addition to studies (n)**					
0 % (103)	59 (6)	46 (13)	34 (7)	24 (6)	22 (4)
1 – 20 % (399)	60 (6)	46 (11)	35 (7)	24 (5)	22 (3)
21 – 50 % (179)	59 (6)	48 (10)	35 (7)	24 (5)	22 (4)
> 50 % (23)	58 (7)	49 (15)	37 (8)	26 (8)	22 (3)
p value*	0.4	0.2	0.5	0.3	0.9
**Age (n)*** **					
<24 years (354)	59 (6)	46 (11)	35 (7)	24 (5)	22 (3)
≥24 years (358)	60 (6)	48 (12)	35 (7)	24 (6)	22 (4)
p value**	0.3	0.002	0.6	1	0.3

**Analysed by factorial ANOVA*
*** Analysed by independent t-test*
****Median value of 24 years defined the middle value for the age groups*
[abc] Groups with the same subscript are significantly different to each other within the same domain. For example, occupational therapists scored significantly higher than radiographers and nurses for Relevance, while physiotherapy scored significantly higher than the radiographers did.
