# Supplementary material for: Evidence-based practice profiles among bachelor students in four health disciplines: a cross-sectional study
Source: BMC Med Educ. 2018 Sep 14;18:210. doi: 10.1186/s12909-018-1319-7 (PMC6137748; doi:10.1186/s12909-018-1319-7)
Supplement: Supplementary file 3 — Estimated differences in mean EBP2-N domain scores between health disciplines (n = 707). The table provides differences in mean EBP2-N domain scores estimated by simple and multiple linear regression for health disciplines with nurses as the reference group. Goodness-of-fit was assessed by the adjusted coefficient of determination (R2). (PDF 74 kb) [file 12909_2018_1319_MOESM3_ESM.pdf]

Additional file 3. Estimated differences in mean EBP^2-N domain scores between health disciplines (n=707).

	Relevance		Terminology		Confidence		Practice		Sympathy	
	Univariate B (CI 95%) P	Multivariate* B (CI 95%) p	Univariate B (CI 95%) p	Multivariate* B (CI 95%) p	Univariate B (CI 95%) p	Multivariate* B (CI 95%) p	Univariate B (CI 95%) p	Multivariate* B (CI 95%) p	Univariate B (CI 95%) p	Multivariate* B (CI 95%) p
**Occupational therapy**	1.6 (0.4 - 2.8) 0.008	1.3 (0.1 – 2.4) 0.04	-2.1 (-4.3 - 0.1) 0.06	-2.8 (-4.9 - -0.7) 0.01	-3.1 (-4.5- -1.7) <0.001	-3.3 (-4.7 - -1.8) <0.001	0.7 (-0.4 – 1.7) 0.2	0.7 (-0.3 – 1.8) 0.2	0.5 (-0.1 – 1.2) 0.1	0.1 (-0.4 - 0.9) 0.5
**Physiotherapy**	0.4 (-1.0 -1.8) 0.6	0.8 (-0.6-2.2) 0.3	5.1 (2.5 – 7.6) <0.001	5.0 (2.5 – 7.5) <0.001	-2.5 (-4.1 - -1.0) 0.002	-2.6 (-4.3 - -0.9) 0.003	-0.8 (-2.0 - 0.4) 0.2	-0.6 (-1.9 – 0.7) 0.4	0.6 (-0.2 – 1.3) 0.2	0.4 (-0.4 – 1.2) 0.3
**Radiography**	-4.9 (-6.6 - -3.1) <0.001	-4.7 (-6.4 - -3.0) <0.001	-1.8 (-4.9 – 1.4) 0.3	-2.9 (-6.0 – 0.2) 0.06	-2.7 (-4.7 - -0.7) 0.009	-3.2 (-5.2 - -1.2) 0.002	-2.5 (-4.0 - -0,9) 0.002	-2.5 (-4.1 - -1.0 0.001	-1.0 (-1.9 – 0.0) 0.05	-1.3 (-2.3 - -0.3) 0.01
**Nurse**	0[a]	0[a]	0[a]	0[a]	0[a]	0[a]	0[a]	0[a]	0[a]	0[a]
**Adjusted R^2**	0.057	0.122		0.119		0.042		0.021		0.041

*adjusted for educational institution, gender, age, previous bachelor education, and work in addition to studies.
