# Supplementary material for: Evidence-based practice profiles among bachelor students in four health disciplines: a cross-sectional study
Source: BMC Med Educ. 2018 Sep 14;18:210. doi: 10.1186/s12909-018-1319-7 (PMC6137748; doi:10.1186/s12909-018-1319-7)
Supplement: Supplementary file 4 — The relationship between EBP2-N domains, assessment of EBP teaching and expectation of EBP performance. The table provides the results of the relationship of the five EBP2-N domains, and students’ assessment of EBP teaching and students’ assumed expectations from teachers of EBP performance, respectively, estimated by Spearman’s rho (rS). (PDF 314 kb) [file 12909_2018_1319_MOESM4_ESM.pdf]

Additional file 4. The relationship between EBP^2-N domains, assessment of EBP teaching and expectation of EBP performance.

Domain	Assessment of EBP teaching			Expectation of EBP performance		
	r_s	n	p	r_s	n	p
**Relevance**	0.307	693	<0.001	0.356	696	<0.001
**Terminology**	0.257	693	<0.001	0.111	695	0.003
**Confidence**	0.460	691	<0.001	0.192	694	<0.001
**Practice**	0.269	690	<0.001	0.278	692	<0.001
**Sympathy**	0.211	688	<0.001	0.183	691	<0.001
